# Magnetoacoustic Waves and the Kelvin–Helmholtz Instability in a Steady Asymmetric Slab: I: The Effects of Varying Density Ratios

**DOI:** 10.1007/s11207-018-1305-6

**Published:** 2018-05-28

**Authors:** M. Barbulescu, R. Erdélyi

**Affiliations:** 1 Solar Physics and Space Plasma Research Centre (SP2RC), School of Mathematics and Statistics, University of Sheffield, Hounsfield Road, Hicks Building, Sheffield, S3 7RH UK; 2 Department of Astronomy, Eötvös Loránd University, Pázmány P. sétány 1/A, Budapest, 1117 Hungary

**Keywords:** MHD waves, Kelvin–Helmholtz instability

## Abstract

Recent observations have shown that bulk flow motions in structured solar plasmas, most evidently in coronal mass ejections (CMEs), may lead to the formation of Kelvin–Helmholtz instabilities (KHIs). Analytical models are thus essential in understanding both how the flows affect the propagation of magnetohydrodynamic (MHD) waves, and what the critical flow speed is for the formation of the KHI. We investigate both these aspects in a novel way: in a steady magnetic slab embedded in an asymmetric environment. The exterior of the slab is defined as having different equilibrium values of the background density, pressure, and temperature on either side. A steady flow and constant magnetic field are present in the slab interior. Approximate solutions to the dispersion relation are obtained analytically and classified with respect to mode and speed. General solutions and the KHI thresholds are obtained numerically. It is shown that, generally, both the KHI critical value and the cut-off speeds for magnetoacoustic waves are lowered by the external asymmetry.

## Introduction

**Figure 1:**
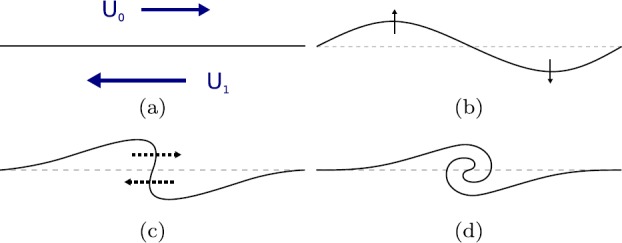
Stages of a Kelvin–Helmholtz instability (KHI). We assume that a magnetic interface (**a**) separating two regions with background flows in opposite directions is subject to a perturbation (**b**). As the system evolves in time, sufficiently strong flows will amplify the perturbation, causing nonlinear wave steepening (**c**), until vortex formation occurs (**d**). Further evolution typically renders the system turbulent.

The propagation of linear magnetohydrodynamic (MHD) waves along magnetic slabs has long been a topic of study in the context of solar physics (see, *e.g.* Roberts, 1981). The presence of a steady flow in the equilibrium state of the system affects the propagation in at least two important ways. First, perturbations may cause shearing motions in the flow, which then could lead to the Kelvin–Helmholtz instability (KHI) (see Figure 1). Second, the phase speeds and the cut-off speeds of each mode of propagation are shifted proportional to the speed of the flow (see, *e.g*. Nakariakov and Roberts, 1995). Interactions between propagating waves and flows are not limited to these two instances, however. Other areas of study include negative-energy wave instabilities, if dissipative effects are taken into account (Cairns, 1979; Joarder, Nakariakov, and Roberts, 1997), or resonant flow instabilities, if resonant wave excitation is considered (see Tirry *et al.*, 1998; Taroyan and Erdélyi, 2002). More information on the above topics may be found in Taroyan and Ruderman (2011) and Ryutova (2015).

The effects of steady flows have been investigated in a number of different waveguide geometries and magnetic topologies. Nakariakov and Roberts (1995) studied the effect of a steady flow in an infinite slab of magnetised plasma in a magnetic environment. Terra-Homem, Erdélyi, and Ballai (2003) then explored the effects that a steady flow has on the propagation of both linear and nonlinear waves in a straight infinite cylindrical flux tube. This latter work expanded on the analysis of Somasundaram, Venkatraman, and Sengottuvel (1999). For a more general approach to analysing the stability of steady MHD flows, see, for example, Goedbloed (2009a,b).

More recently, Soler *et al.* (2010) described the effects of an azimuthally dependent flow on the stability of a straight flux tube, while Zaqarashvili, Vörös, and Zhelyazkov (2014) investigated the stability of an incompressible, twisted cylindrical flux tube, subject to a straight flow, in a magnetic environment. Finally, Zaqarashvili, Zhelyazkov, and Ofman (2015) studied the stability of an incompressible, rotating, and twisted cylinder. The theoretical results of the latter two works were applied in Kuridze *et al.* (2016) to determine the stability of chromospheric jets, and to estimate the growth time of the KHI.

Recent observational results have reinforced the idea that plasma flows are present throughout the solar atmosphere. Berger *et al.* (2010) and Ryutova *et al.* (2010) uncovered details about mass flows and the formation of the KHI in solar prominences. KHI formation in the corona has also received considerable attention (see Foullon *et al.*, 2011, 2013; Ofman and Thompson, 2011). For a recent review, see Zhelyazkov (2015).

Of significant interest are the observations by Foullon *et al.* (2011) of a KHI on the flank of a CME. The authors interpret the system configuration as consisting of three regions: the dense solar ejecta, the CME sheath, and the low-density corona, with the KHI occurring in the region between the ejecta sheath and the corona. A similar three-layer system is described by Möstl, Temmer, and Veronig (2013). By interpreting the CME boundary as a steady magnetic slab embedded in an asymmetric magnetic environment, the authors demonstrated that through increasing the magnetic field strength on only one side of the slab, the field provided a stabilising effect on that side only. This numerical study shows that exterior asymmetry may be an important factor when considering the physics of magnetic slabs.

The three-layer system, envisioned as a slab in an asymmetric environment, has recently been studied by Allcock and Erdélyi (2017) and Zsámberger, Allcock, and Erdélyi (2018) in the context of linear wave propagation. Here, we focus on the effects that a steady flow within the slab has on the propagation of magnetoacoustic waves, and on how the asymmetry affects the KHI threshold values. In Section 2, we assume that our system is governed by the ideal MHD equations, and we derive the dispersion relation for waves propagating along the slab. In Section 3, we obtain approximate solutions to the dispersion relation in the thin slab limit, and classify the modes in terms of the characteristic speeds of the system. In Section 4, we obtain general solutions to the dispersion relation and also the KHI thresholds. Finally, Section 5 summarises the results and provides context for their implications.

## The Dispersion Relation

**Figure 2:**
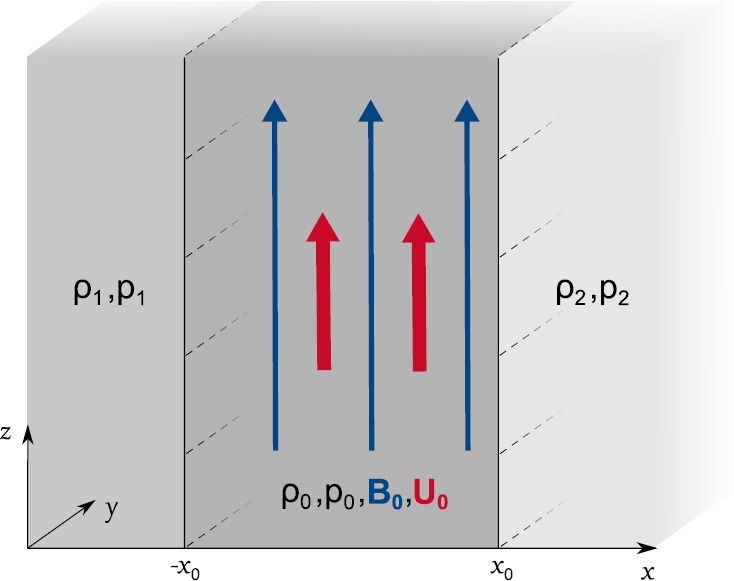
Steady magnetic slab embedded in a static asymmetric unmagnetised environment.

We introduce a slab of plasma bounded by two interfaces at $\pm x_{0}$, of density, pressure, and temperature $\rho_{0}$, $p_{0}$, and $T_{0}$, respectively, and magnetic field $\mathbf{B}_{0} = (0, 0, B _{0})$, which is subject to a steady flow $\mathbf{U}_{0} = (0, 0, U _{0})$. The slab is embedded in an asymmetric environment, defined as having density, pressure, and temperature $\rho_{1}$, $p_{1}$, and $T_{1}$ on the left side, and $\rho_{2}$, $p_{2}$, and $T_{2}$ on the right side, as illustrated in Figure 2. The exterior is neither subject to magnetic fields nor to flows. It follows that the fluid in the interior region of the slab is governed by the ideal MHD equations, while the exterior regions are described using the gas equations.

We wish to obtain a governing equation describing the propagation of linear magnetoacoustic waves along the parallel interfaces. Linearising the ideal MHD equations, subject to the previously defined background conditions, allows us to write them in the form

1 $$\begin{aligned} \begin{aligned} \frac{\mathrm{D} \rho}{\mathrm{D} t} + \rho_{0} \nabla\cdot \mathbf{v} &= 0, \\ \rho_{0}\frac{\mathrm{D} \mathbf{v}}{\mathrm{D} t} &= - \nabla\biggl( p + \frac{1}{\mu} b_{z} B_{0} \biggr) + \frac{B_{0}}{\mu} \frac{\partial \mathbf{b}}{\partial z}, \\ \frac{\mathrm{D} p}{\mathrm{D} t} &= c_{0}^{2} \frac{\mathrm {D}\rho}{\mathrm{D} t}, \\ \frac{\mathrm{D} \mathbf{b}}{\mathrm{D} t} &= - \mathbf{B}_{0} (\nabla\cdot\mathbf{v} )+ B_{0} \frac{\partial\mathbf {v}}{\partial z}. \end{aligned} \end{aligned}$$

Here $\rho, p, \mathbf{b} = (b_{x}, b_{y}, b_{z})$, and $\mathbf{v} = (v_{x}, v_{y}, v_{z})$ are small perturbations from the equilibrium, and $\frac{\mathrm{D}}{\mathrm{D} t} = \frac{\partial}{\partial t} + U _{0} \frac{\partial}{\partial z}$ is the material derivative. The sound speed is defined as $c_{0}^{2} = \gamma p_{0}/\rho_{0}$.

Since we are only concerned with magnetoacoustic waves, we may disregard all dependence on the $y$-component without loss of generality. Equations 1 may, thus, be written in component form as

2 $$\begin{aligned} \begin{aligned} \rho_{0} \frac{\mathrm{D} v_{x}}{\mathrm{D} t} & = - \frac{\partial}{\partial x} \biggl( p + \frac{B_{0}}{\mu_{0}} b_{z} \biggr) + \frac{B_{0}}{\mu_{0}} \frac{\partial b_{x}}{\partial z}, \\ \rho_{0} \frac{\mathrm{D} v_{z}}{\mathrm{D} t} & = - \frac{\partial p}{\partial z}, \\ \frac{\mathrm{D} p}{\mathrm{D} t} & = - c_{0}^{2} \rho_{0} \nabla\cdot\mathbf{v}, \\ \frac{\mathrm{D} b_{x}}{\mathrm{D} t} & = B_{0} \frac{\partial v_{x}}{\partial z}, \\ \frac{\mathrm{D} b_{z}}{\mathrm{D} t} & = - B_{0} \frac{\partial v_{x}}{\partial x}. \end{aligned} \end{aligned}$$

We Fourier-decompose Equations 2 for waves propagating along the slab by assuming that $f(\mathbf{r}, t) = \hat{f}(x) \mathrm{e}^{-\mathrm{i} (\omega t - k z)}$, where $f$ stands for any of the small perturbations, and $\hat{f}$ is the amplitude of each perturbation. Here, $\omega$ is the angular frequency, and $k$ is the wavenumber in the $z$-direction. This procedure allows us to remove all differential terms in the linearised MHD equation, except for derivatives with respect to $x$. Equations 2 become

3 $$\begin{aligned} \begin{aligned} i \rho_{0} \Omega \hat{v}_{x} & = \frac{\mathrm{d}}{\mathrm{d} x} \biggl( \hat{p} + \frac{B_{0}}{\mu_{0}} \hat{b}_{z} \biggr) + ik \frac{B _{0}}{\mu_{0}} \hat{b}_{x}, \\ \rho_{0} \Omega\hat{v}_{z} & = k \hat{p}, \\ \Omega\hat{p} & = c_{0}^{2} \rho_{0} \biggl(- i \frac{\mathrm{d} \hat{v} _{x}}{\mathrm{d} x} + k \hat{v}_{z}\biggr), \\ \Omega\hat{b}_{x} & = - B_{0} k \hat{v}_{x}, \\ i \Omega\hat{b}_{z} & = B_{0} \frac{\mathrm{d} \hat{v}_{x}}{ \mathrm{d} x}, \end{aligned} \end{aligned}$$

where $\Omega= \omega- k U_{0}$ is the Doppler-shifted frequency.

Equations 3 may be manipulated such that, except for $\hat{v}_{x}$, all other perturbed quantities are eliminated, leaving us with the governing equation for the velocity amplitude:

4 $$ \hat{v}_{x}'' - m_{0}^{2} \hat{v}_{x} = 0, \quad m_{0}^{2} = \frac{ ( k^{2} v_{\mathrm{A}}^{2} - \Omega^{2} ) ( k^{2} c_{0}^{2} - \Omega^{2}) }{ ( c_{0}^{2} + v_{\mathrm{A}}^{2} ) ( k^{2} c_{\mathrm{T}}^{2} - \Omega ^{2} )}, $$

where the Alfvén speed $v_{\mathrm{A}}$ and tube speed $c_{\mathrm{T}}$ are defined as

$$ v_{\mathrm{A}}^{2} = \frac{B_{0}^{2}}{\mu_{0} \rho_{0}}, \quad c_{\mathrm{T}}^{2} = \frac{c _{0}^{2} v_{\mathrm{A}}^{2}}{c_{0}^{2} + v_{\mathrm{A}}^{2}}. $$

The same scheme may be applied to the exterior layers, with the consideration that in both semi-infinite layers, there are no magnetic fields or flows present. The governing equations for the outer layers are thus

5 $$ \hat{v}_{x}'' - m_{j}^{2} \hat{v}_{x} = 0, \qquad m_{j}^{2} = k^{2} - \frac{\omega^{2}}{c_{j}^{2}}, \quad \mbox{for } j = 1, 2, $$

where the exterior sound speeds are defined as $c_{j}^{2} = \gamma p _{j}/\rho_{j}$.

We find trapped wave solutions to Equations 4 and 5. For the solutions to Equations 5 to be realistic, they need to be evanescent (*i.e.* all perturbations must vanish at $\pm \infty$), meaning that $m_{j}^{2} > 0$ is required for $j = 1, 2$. This yields the general solution of Equations 4 and 5

6 $$ \hat{v}_{xj} (x) = \textstyle\begin{cases} A(\cosh m_{1} x + \sinh m_{1} x), & x < - x_{0}, \\ B \cosh m_{0} x + C \sinh m_{0} x, & |x| \leq x_{0}, \\ D(\cosh m_{2} x - \sinh m_{2} x), & x > x_{0}, \\ \end{cases} $$

where $A$, $B$, $C$, and $D$ are arbitrary constants. By inspection, we establish that two wave modes are allowed to propagate under the given constraints: one that is evanescent towards the centre of the slab (for $m_{0}^{2} > 0$), and one that is spatially oscillatory throughout the slab (for $m_{0}^{2} < 0$). These modes of propagation are the so-called surface and body modes, respectively (see, *e.g.* Roberts, 1981).

Equation 6 is subject to boundary conditions at the interfaces, namely, the continuity of the Lagrangian displacement, and the continuity of total pressure:

7 $$\begin{aligned} \begin{aligned} & \frac{\hat{v}_{x1} (x = - x_{0})}{\omega} = \frac{\hat{v}_{x0} (x = - x_{0})}{\Omega}, \\ & \frac{\hat{v}_{x2} (x = x_{0})}{\omega} = \frac{\hat{v}_{x0} (x = x_{0})}{\Omega}, \\ & [p_{\mathrm{T}}]_{-x_{0}} = 0, \quad \quad [p_{\mathrm{T}}]_{x_{0}} = 0, \end{aligned} \end{aligned}$$

where the total pressure is defined as

8 $$ \hat{p}_{\mathrm{T}} (x) = \hat{v}'_{xj} (x) \textstyle\begin{cases} \dfrac{i \rho_{1} \omega}{m_{1}^{2}}, & x < - x_{0}, \\ -\dfrac{i \rho_{0} (k^{2} v_{\mathrm{A}}^{2} - \Omega^{2})}{m_{0}^{2} \Omega }, & |x| \leq x_{0}, \\ \dfrac{i \rho_{2} \omega}{m_{2}^{2}}, & x > x_{0}. \\ \end{cases} $$

Using Equation 6 and the associated boundary conditions 7 and 8, we obtain a system of four coupled homogeneous algebraic equations

9 $$ \begin{pmatrix} c_{1} - s_{1} & - c_{0} \omega/\Omega& s_{0} \omega/\Omega& 0 \\ 0 & c_{0} \omega/\Omega& s_{0} \omega/\Omega& s_{2} - c_{2} \\ \Lambda_{1} (c_{1} - s_{1}) & - \Lambda_{0} s_{0} & \Lambda_{0} c _{0} & 0 \\ 0 & - \Lambda_{0} s_{0} & - \Lambda_{0} c_{0} & \Lambda_{2} (c_{2} - s_{2}) \end{pmatrix} \begin{pmatrix} A \\ B \\ C \\ D \end{pmatrix} = \begin{pmatrix} 0 \\ 0 \\ 0 \\ 0 \end{pmatrix} , $$

where, for brevity, we introduced $c_{j} = \cosh m_{j} x_{0}$, $s_{j} = \sinh m_{j} x_{0}$, for $j = 0,1,2$, and

$$ \Lambda_{0} = \frac{i \rho_{0} ( k^{2} v_{\mathrm{A}}^{2} - \Omega^{2} ) }{m_{0} \Omega}, \qquad \Lambda_{1} = \frac{i \rho_{1} \omega}{m_{1}}, \qquad \Lambda_{2} = \frac{i \rho_{2} \omega}{m_{2}}. $$

For Equation 9 to have non-trivial solutions, we require the determinant of the matrix on the left-hand side to be equal to zero. Evaluating this condition, we obtain

10 $$\begin{aligned} \begin{aligned} (\Lambda_{0}s_{0} - \Lambda_{1} c_{0} \omega/\Omega) (\Lambda_{0} c_{0} - \Lambda_{2} s_{0}\omega/\Omega) + ( \Lambda_{0} c_{0} - \Lambda_{1} s_{0} \omega/\Omega ) ( \Lambda_{0} s_{0} - \Lambda_{2} c_{0} \omega/\Omega ) = 0, \end{aligned} \end{aligned}$$

which, after some algebra, yields the dispersion relation for magnetoacoustic waves in a steady magnetic slab embedded in an asymmetric non-magnetic environment

11 $$\begin{aligned} \begin{aligned} &m_{0}^{2}\omega^{4} + \frac{\rho_{0}}{\rho_{1}} m_{1} \frac {\rho _{0}}{\rho_{2}} m_{2} \bigl( k^{2} v_{\mathrm{A}}^{2} - \Omega^{2} \bigr)^{2} - \frac{1}{2} m_{0} \omega^{2} \bigl( k^{2} v_{\mathrm{A}}^{2} - \Omega^{2}\bigr) \biggl( \frac{\rho_{0}}{\rho_{1}} m_{1} + \frac{\rho_{0}}{\rho_{2}} m_{2} \biggr) \\ &\quad {}\times\bigl( \tanh(m_{0} x_{0}) + \coth(m_{0}x_{0}) \bigr) = 0. \end{aligned} \end{aligned}$$

Equation 11 is a generalisation of the dispersion relations found in Nakariakov and Roberts (1995) and Allcock and Erdélyi (2017). The dispersion relation of Allcock and Erdélyi (2017) may be immediately recovered by removing the background flow, *i.e.* setting $U_{0} = 0$. On the other hand, if we retain the background flow but eliminate the asymmetric density profile (*i.e.* $\rho_{1} = \rho_{2}$), we recover the dispersion relation of Nakariakov and Roberts (1995).

An interesting feature of Equation 11 is that as opposed to similar results obtained by Roberts (1981) and Nakariakov and Roberts (1995), it does not factorise into two separate equations. In these studies, due to the symmetry of the environment, the disturbances may be divided into two modes of oscillation: the sausage mode, where the two interfaces oscillate in anti-phase, and the kink mode, where they oscillate in phase. The amplitude of the velocity perturbation, $\hat{v}_{x}(x)$, is an odd function in the case of a sausage mode, and an even function in the case of a kink mode. Moreover, when factorising the dispersion relation, the equation containing $\tanh(m_{0} x_{0})$ corresponds to the sausage mode, and the one containing $\coth(m_{0} x_{0})$ to the kink mode.

It has been shown by Allcock and Erdélyi (2017) that in a slab embedded in an asymmetric environment, there still exist two classes of modes of oscillation analogous to Roberts (1981). However, due to density asymmetry, the amplitudes of the perturbations on either side of the slab will not be equal, meaning that the eigenfunctions are neither odd nor even. This explains why Equation 11 cannot be factorised: both $\tanh(m_{0} x_{0})$ and $\coth(m_{0} x_{0})$ are required to describe the asymmetric solutions. Allcock and Erdélyi (2017) labelled these asymmetric modes as quasi-sausage, when the perturbations are in anti-phase, and quasi-kink, if they are in phase.

## Mode Classification and Analytical Solutions

Information about the nature of the wave solutions may be obtained from the parameters of the dispersion relation. We have already established that in order for waves to be trapped, the exterior parameters $m_{1}^{2}$ and $m_{2}^{2}$ must be positive. Modes that do not meet this condition are referred to as leaky and are excluded from the analysis in the present work. We define the phase speed as $c_{\mathrm {ph}} = \omega/ k$ and deduce that for modes to be trapped, they must satisfy $\textrm{max}(-c_{1}, -c_{2}) < c_{\mathrm{ph}} < \textrm {min}(c_{1}, c_{2})$. It is also worth noting that the sign of the phase speed, $c_{\mathrm{ph}}$, determines whether modes are forward or backward propagating, a positive sign corresponding to the former and a negative to the latter.

The parameter $m_{0}^{2}$ offers a means of classifying the solutions obtained numerically. We have already established that surface modes satisfy the condition $m_{0}^{2} > 0$, while body modes require $m_{0}^{2} < 0$. We may therefore categorise all solutions of Equation 11 with respect to the signs of $c_{\mathrm{ph}}$, $m_{0}^{2}$, $m_{1} ^{2}$, and $m_{2}^{2}$.

**Figure 3:**
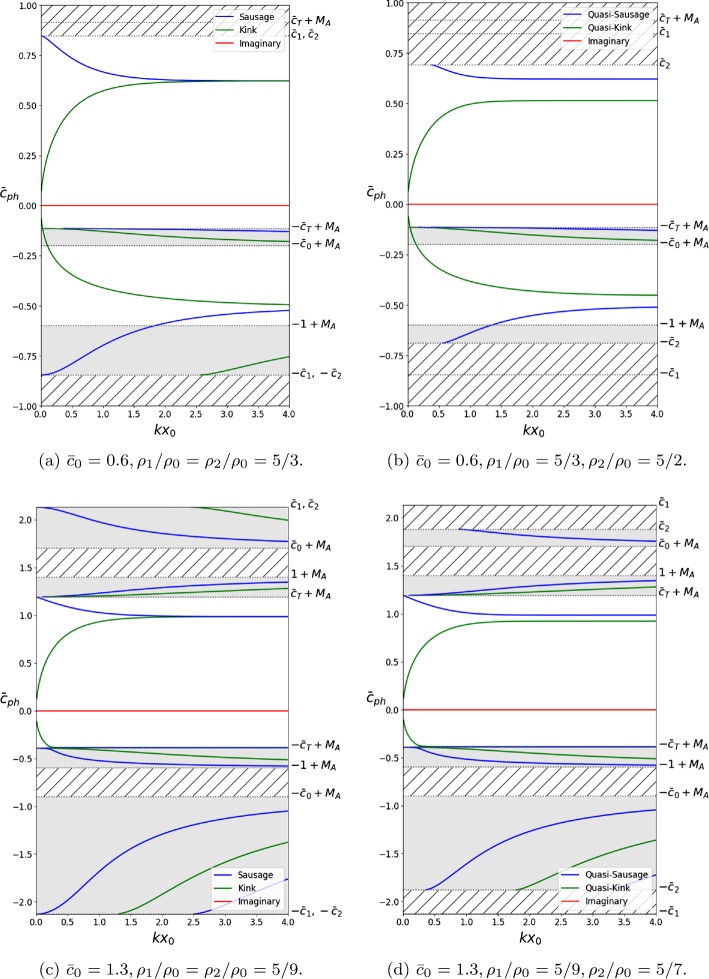
Dispersion diagrams considering an interior that is dense ((**a**) and (**b**)), and one that is evacuated ((**c**) and (**d**)), including a background flow of Alfvén Mach number $M_{\mathrm{A}} = 0.4$. Panels (**a**) and (**c**) illustrate the solutions obtained for symmetric exterior density profiles, while (**b**) and (**d**) illustrate the effects of breaking this symmetry. The asymmetric density profile introduces new cut-off frequencies at min($c_{1}$, $c_{2}$), while the flow further breaks the symmetry by causing forward- ($\bar{c}_{\mathrm{ph}} > 0$) and backward- ($\bar{c}_{\mathrm{ph}} < 0$) propagating modes to have different phase speeds. The *shaded areas* represent regions for which body modes propagate. The *hatched regions* contain no stable trapped solutions ($m_{1}^{2} < 0$ or $m_{2}^{2} < 0$).

Solutions that satisfy $\textrm{max}(c_{0}, v_{\mathrm{A}}) < |c_{\mathrm{ph}} - U_{0}| < \textrm{min}(c_{1} - U_{0}, c_{2} - U_{0})$ are fast surface or body modes, depending on the sign of $m_{0}^{2}$, which is determined by the ordering of the characteristic speeds. Panels (c) and (d) in Figure 3 contain forward-propagating body mode solutions in this interval. However, they are absent from panels (a) and (b) because $\textrm{min}(c_{1}, c_{2}) < \textrm{min}(c_{0} + U_{0}, v_{\mathrm{A}} + U _{0})$. Slow body and surface modes have phase speeds within the interval $c_{\mathrm{T}} < |c_{\mathrm{ph}} - U_{0}| < \textrm{min}(c_{0}, v_{\mathrm{A}})$ and $|c_{\mathrm{ph}} - U_{0}| < c_{\mathrm{T}}$, respectively.

Equation 11 is, to the best of our knowledge, insoluble analytically without the use of simplifying approximations. We thus employ the assumption that the wavelength of the propagating wave solutions is much longer than the width of the slab, *i.e.* that $k x_{0} \ll1$. This also implies that for surface modes, $m_{0} x _{0} \to0$ as $k x_{0} \to0$, and that $\tanh m_{0} x_{0} \approx m _{0} x_{0}$, and $\coth m_{0} x_{0} \approx(m_{0} x_{0})^{-1}$. The dispersion relation, Equation 11, may then be written as

12 $$\begin{aligned} \begin{aligned} & m_{0}^{2}\omega^{4} + \frac{\rho_{0}}{\rho_{1}} m_{1} \frac{\rho_{0}}{\rho_{2}}m_{2} \bigl( k^{2} v_{\mathrm{A}}^{2} - \Omega^{2} \bigr)^{2} \\ &\quad {} - \frac{1}{2} m_{0} \omega^{2} \bigl( k^{2} v_{\mathrm{A}}^{2} - \Omega^{2}\bigr) \biggl( \frac{\rho_{0}}{\rho_{1}} m_{1} + \frac{\rho_{0}}{\rho _{2}} m _{2} \biggr) \biggl( m_{0} x_{0} + \frac{1}{m_{0} x_{0}} \biggr) = 0. \end{aligned} \end{aligned}$$

Following Roberts (1981), we look for surface mode solutions of the form

$$ \omega= \omega_{(0)} + k x_{0} \omega_{(1)} + \mathcal{O}\bigl(k^{2} x _{0}^{2}\bigr). $$

By taking the terms of order $k x_{0}$ in Equation 12, we find the first-order terms in the perturbation expansion, and hence obtain two solutions: one for the Doppler-shifted quasi-sausage mode with $\Omega^{2} \approx k^{2} c_{\mathrm{T}}^{2}$:

13 $$ \Omega^{2} \approx k^{2} c_{\mathrm{T}}^{2} \biggl( 1 - 2 k x_{0} \frac{(c_{0} ^{2} - c_{\mathrm{T}}^{2}) (c_{\mathrm{T}} + U_{0})^{2}}{ (c_{0}^{2} + v_{\mathrm{A}}^{2}) c_{\mathrm{T}} ^{2} [ \frac{\rho_{0}}{\rho_{1}} \frac{(c_{1}^{2} - (c_{\mathrm{T}} + U _{0})^{2})^{1/2}}{c_{1}} + \frac{\rho_{0}}{\rho_{2}} \frac{(c_{2}^{2} - (c_{\mathrm{T}} + U_{0})^{2})^{1/2}}{c_{2}} ] } \biggr) , $$

and one for its companion quasi-kink mode with $\omega^{2} \to0$ as $k x_{0} \to0$:

14 $$ \omega^{2} \approx k x_{0} \frac{2 \rho_{0}}{\rho_{1} + \rho_{2}} \bigl(k ^{2} v_{\mathrm{A}}^{2} - k^{2} U_{0}^{2}\bigr). $$

Roberts (1981) also found a surface sausage mode solution with $\omega^{2} \approx k^{2} c_{\mathrm{e}}^{2}$, but this solution no longer exists unless a single exterior sound speed $c_{1} = c_{2} = c_{\mathrm{e}}$ exists.

In order to find body mode solutions, we must be aware that our previous assumption, that $m_{0} x_{0} \to0$ as $k x_{0} \to0$, no longer holds. Instead, we must find solutions for which $m_{0} x_{0}$ is non-zero and finite as $k x_{0}$ tends to zero. We are interested in solutions with $\Omega^{2} \approx k^{2} c_{\mathrm{T}}^{2}$ and $m_{0}^{2} < 0$. From Equation 11 we obtain two solutions, one describing the behaviour of the Doppler-shifted quasi-sausage modes,

15 $$ \Omega^{2} \approx k^{2} c_{\mathrm{T}}^{2} \biggl( 1 + k^{2} x_{0}^{2} \dfrac{(v _{\mathrm{A}}^{2} - (c_{\mathrm{T}} - U_{0})^{2} )(c_{0}^{2} - (c_{\mathrm{T}} - U_{0})^{2} ) }{c _{0}^{2} v_{\mathrm{A}}^{2} \pi^{2} j^{2}} \biggr) , $$

and one describing the set of Doppler-shifted quasi-kink modes,

16 $$ \Omega^{2} \approx k^{2} c_{\mathrm{T}}^{2} \biggl( 1 + k^{2} x_{0}^{2} \dfrac{(v _{\mathrm{A}}^{2} - (c_{\mathrm{T}} - U_{0})^{2} )(c_{0}^{2} - (c_{\mathrm{T}} - U_{0})^{2} ) }{c _{0}^{2} v_{\mathrm{A}}^{2} \pi^{2} (j- \frac{1}{2})^{2}} \biggr) , $$

where $j$ is any integer.

In the case of a wide slab, *i.e.* when the slab width is much larger than the wavelength, we demonstrate that the two interfaces that delimit the slab cease interacting. We begin by taking $k x_{0} \gg1$, which implies that for surface modes, $m_{0} x_{0} \gg1$ (Roberts, 1981). In this approximation, $\tanh(m_{0} x_{0}) \approx\coth(m_{0} x_{0}) \approx1$, which, when applied to Equation 10, provides us with two individual dispersion relations for the two interfaces

17 $$\begin{aligned} \begin{aligned} \frac{\rho_{0}}{\rho_{j}} m_{j} \bigl( k^{2} v_{\mathrm{A}}^{2} - \Omega^{2} \bigr) - m_{0} \omega^{2} = 0, \end{aligned} \end{aligned}$$

for $j=1,2$.

Equations 13 to 17 may be reduced to the analogous equations in Allcock and Erdélyi (2017) by setting $U_{0} = 0$, and to those in Roberts (1981) by also assuming that $\rho_{1} = \rho_{2} = \rho_{\mathrm{e}}$.

## Numerical Results

We now find the general solutions to the dispersion relation, Equation 11. Since, to the best of our knowledge, these cannot be obtained analytically, we employ a numerical scheme. We first nondimensionalise all quantities with respect to the Alfvén speed, and introduce the Alfvén Mach number $M_{\mathrm{A}} = U_{0}/v_{\mathrm{A}}$, the nondimensionalised sound speeds $\bar{c}_{j}^{2} = c_{j}^{2} / v_{\mathrm{A}} ^{2}$ (for $j = 0, 1, 2$), tube speed $\bar{c}_{\mathrm{T}}^{2} = c_{\mathrm{T}}^{2} / v _{\mathrm{A}}^{2}$, and phase speed $\bar{c}_{\mathrm{ph}} = c_{\mathrm{ph}} / v_{\mathrm{A}} = \omega/ k v_{\mathrm{A}}$.

**Figure 4:**
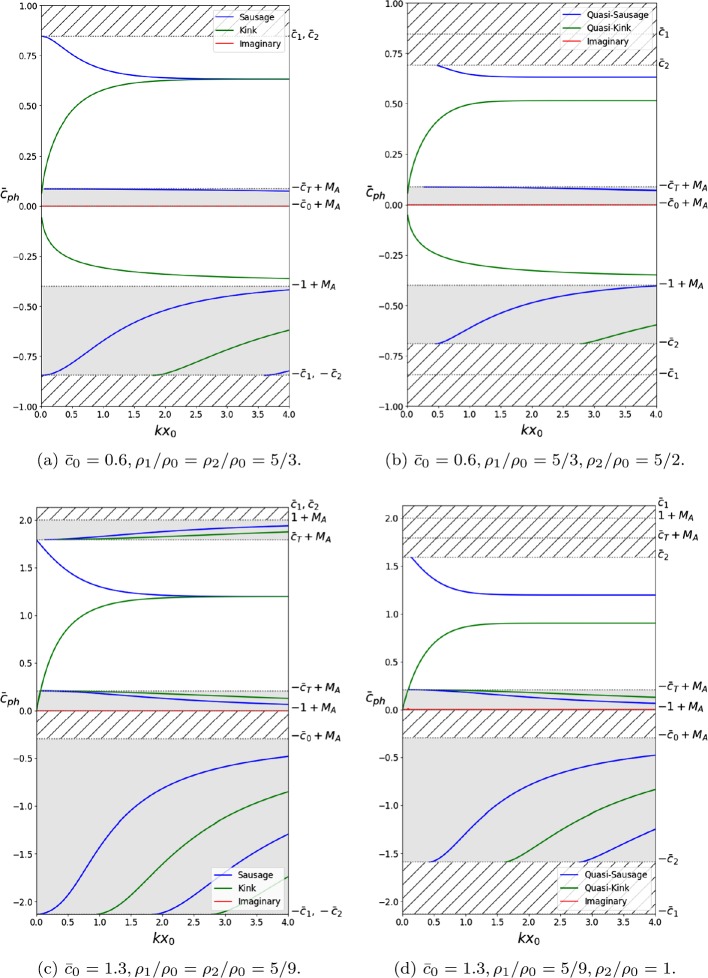
Same as Figure 3, but including background flows of Alfvén Mach number $M_{\mathrm{A}} = 0.6$ ((**a**) and (**b**)), and $M_{\mathrm{A}} = 1.0$ ((**c**) and (**d**)). The bulk flow is now strong enough to have caused the backward-propagating slow body modes to become forward propagating. The asymmetric density profile does not affect the threshold value at which this happens.

**Figure 5:**
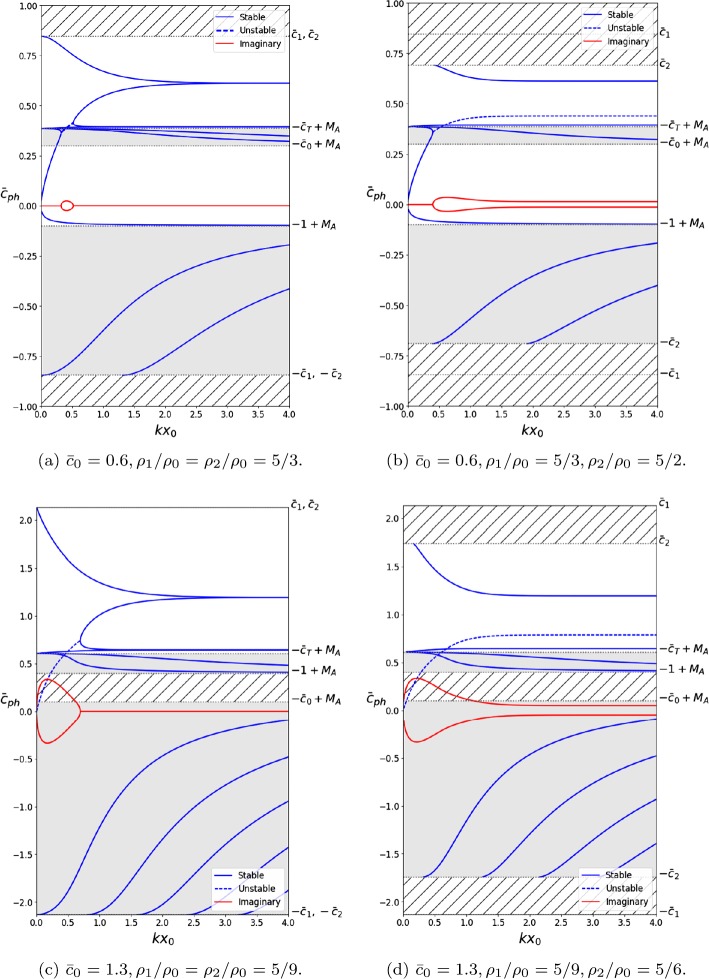
Same as Figure 3, but including background flows of Alfvén Mach number $M_{\mathrm{A}} = 0.9$ ((**a**) and (**b**)), and $M_{\mathrm{A}} = 1.4$ ((**c**) and (**d**)). In the symmetric case ((**a**) and (**c**)), the KHI occurs for a small interval of $k x_{0}$. If the exterior density profile is sufficiently asymmetric, the sausage mode becomes KH unstable for any value of $k x_{0}$ greater than the threshold value.

Dispersion diagrams displaying general solutions to Equation 11 may be found in Figures 3 to 5. They illustrate the behaviour of surface and body, quasi-sausage and quasi-kink modes, under the effect of a number of different flow speeds. Four types of equilibrium conditions are assumed for the slab, each of which is represented in a panel in Figures 3 to 5, respectively. Panels (a) and (b) represent the case where $c_{\mathrm{T}} < c _{0} < v_{\mathrm{A}}$ and the density inside the slab is greater than that of the exterior. Panels (c) and (d) represent the case where $v_{\mathrm{A}} < c _{\mathrm{T}} < c_{0}$ and the exterior densities are greater.

In order to better visualise the differences between the symmetric and asymmetric environments, we have included side-by-side phase diagrams that illustrate the change in behaviour due to the break in symmetry. Thus, in every Figure, panels (a) and (c) depict symmetric exterior profiles, while panels (b) and (d) represent asymmetric exterior profiles.

**Figure 6:**
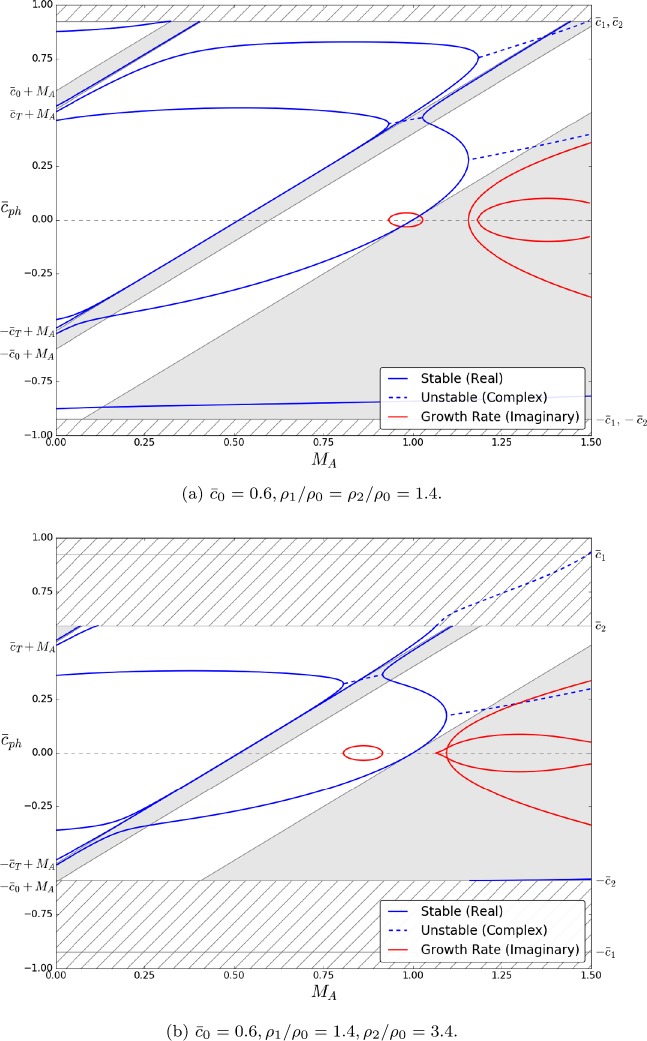
Nondimensionalised phase speed $\bar{c}_{\mathrm{ph}}$ plotted with respect to the Alfvén Mach number $M_{\mathrm{A}}$ for $k x_{0} = 0.5$. The *shaded areas* represent regions where body modes propagate. The *hatched regions* contain no stable trapped solutions ($m_{1}^{2} < 0$ or $m_{2}^{2} < 0$), but unstable solutions may still exist because they have both real and imaginary components. Increasing the density on just one side of the slab decreases the KH threshold and lowers cut-off speeds. Thus, there may exist ranges of $M_{\mathrm{A}}$ where trapped modes, which would otherwise be able to propagate, become leaky.

The imaginary part of the solutions to Equation 11 is displayed throughout Figures 3 to 6 in order to make a distinction between stable and unstable modes. Stable modes correspond to purely real solutions, while unstable modes will have a non-zero imaginary component that will act as a growth factor since we assumed that all perturbations are proportional to $\mathrm{e}^{-\mathrm{i} (\omega t - k z)}$.

Figure 3 illustrates how a background flow of $M_{\mathrm{A}} = 0.4$ affects the phase diagrams in all four cases. We observe that this flow speed has broken the symmetry between forward- and backward-propagating solutions in all cases. Moreover, new cut-off speeds at $\mbox{min}(c_{1}, c _{2})$ have been introduced by the asymmetric exterior profiles (panels (b) and (d)).

Figure 4 displays the effects of a background flow of $M_{\mathrm{A}} = 0.6$ in panels (a) and (b) and $M_{\mathrm{A}} = 1.0$ in panels (c) and (d). These flow strengths are strong enough to cause the slow body modes, which would have been backward propagating for lesser speeds, to now become forward propagating. Different flow strengths are required depending on how the characteristic speeds are ordered. We note that the behaviour is identical throughout the four panels, meaning that the asymmetry in the equilibrium profiles does not affect the change in direction with increasing $M_{\mathrm{A}}$.

Figure 5 illustrates the behaviour of the system subject to a flow of $M_{\mathrm{A}} = 0.9$ in panels (a) and (b), and $M_{\mathrm{A}} = 1.4$ in panels (c) and (d), which is strong enough for instabilities to occur. We see that in the case of symmetric equilibrium profiles, the instability is restricted to a short range of values of $k x_{0}$. However, if the exterior parameters are asymmetric, the mode that was previously unstable in only that small interval is now unstable for any value of $k x_{0}$ greater than the instability onset value.

One important point to note here is that Figures 3 – 5 reinforce the results obtained in Section 3. It is readily visible that for $k x_{0} \ll1$, there exist Doppler-shifted quasi-sausage and quasi-kink surface modes with phase speeds approximately equal to $\bar{c}_{\mathrm{T}} + M_{\mathrm{A}}$ and 0, respectively. Likewise, there exist body modes with $\bar{c}_{\mathrm{ph}} \approx\pm\bar{c}_{\mathrm{T}} + M_{\mathrm{A}}$. The quasi-sausage mode is only present in Figure 3(c) because of the ordering of the characteristic speeds, while the rest are present throughout.

In Figure 6, the phase speed has been plotted with respect to $M_{\mathrm{A}}$, for $k x_{0} = 0.5$ and two different density ratios. The values of the density ratios and of $k x_{0}$ were selected in order to have a clear representation of the modes in the figures. However, the chosen value of $k x_{0}$ may also be relevant to modelling instabilities on CME flanks, for example. Using the values of the wavelengths of the unstable perturbation and the width of shear layer as measured by, *e.g.*, Foullon *et al.* (2011), we obtain a possible range for $k x_{0}$ between 0.131 and 0.656. Panel (a) represents a symmetric density profile, panel (b) an asymmetric one, and both satisfy $c_{\mathrm{T}} < c_{0} < v_{\mathrm{A}}$. Comparing the two panels, it is immediately apparent that by increasing $\rho_{2}$, both the cut-off at $\bar{c}_{2}$ and the KHI threshold are lowered. It is also worth noting that in panel (b), modes with $c_{\mathrm{ph}} > \min(c_{1}, c_{2})$ may exist as long as they are unstable since they satisfy the condition that $c_{\mathrm{ph}}^{2} > \textrm{min}(c_{1}^{2}, c_{2}^{2})$ and are thus trapped. There also exist no fast modes because $\min(\bar{c}_{1}, \bar{c} _{2}) > \min(\bar{c}_{0} + M_{\mathrm{A}}, 1 + M_{\mathrm{A}})$.

**Figure 7:**
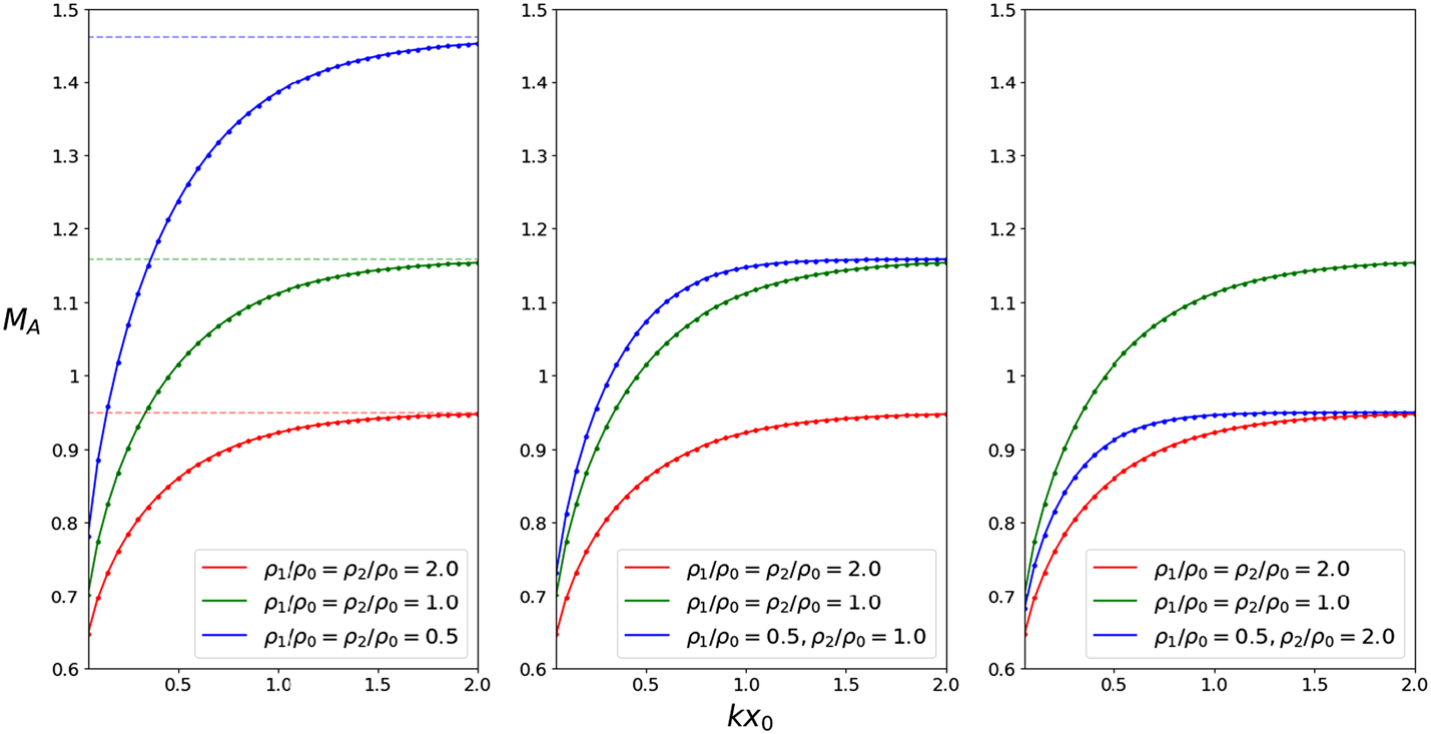
KHI threshold values of $M_{\mathrm{A}}$, calculated for values of $k x_{0}$ from 0.05 to 2, for symmetric and asymmetric density profiles. The *dashed lines* represent the threshold values of a single interface and correspond to the density ratios of their respective colour.

Figure 7 showcases the effect of having an asymmetric density profile on the KHI threshold value. Throughout the panels, the green and red curves (plotted for $\rho_{1} = \rho_{2} = \rho_{0}$ and $\rho_{1} = \rho_{2} = 2 \rho_{0}$, respectively) represent the symmetric density profiles. In the left panel, the blue curve also represents a symmetric density profile, corresponding to a lower density ratio of $\rho_{1} = \rho_{2} = 0.5 \rho_{0}$. This panel illustrates how, for symmetric density profiles, the KHI threshold increases, both with increasing values of $k x_{0}$, but also with decreasing values of the density ratios. As suggested by Equation 17, the threshold value for a wide slab tends to that of a single interface. The middle and right panels illustrate the effect of increasing asymmetry in the density ratios. Owing to the lack of interaction between the interface when $k x_{0} \gg1$, the greater density ratio will determine the threshold value. However, if $k x_{0} \lessapprox1$, the densities on either side will play a role.

**Figure 8:**
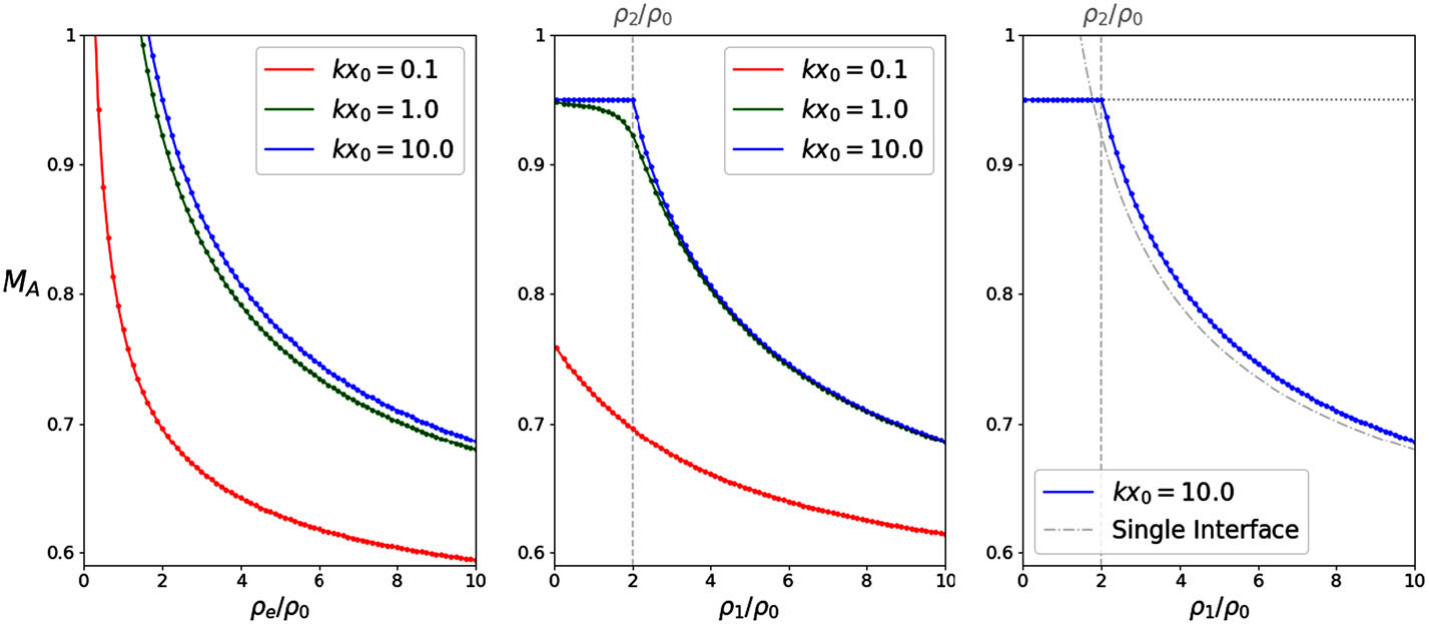
KHI threshold values of $M_{\mathrm{A}}$, calculated for symmetric (*left*) and asymmetric density profiles (*centre*, $\rho_{2}/\rho_{0}=2$). The *panel* on the *right* compares the threshold values obtained for the wide asymmetric slab to that of two non-interacting interfaces. The *dotted horizontal line* and the *dot-dashed curve* represent the threshold values for the interfaces with constant and variable density ratios, respectively.

Figure 8 compares the effects of increasing density ratios in the case of symmetric (left) and asymmetric slabs (centre). In both cases, three slab widths are considered: a thin slab (red), with $k x_{0} = 0.1$, an “intermediate” value of $k x_{0} = 1$ (green), and a wide slab (blue), with $k x_{0} = 10$. In the left panel, the exterior densities are assumed to be equal ($\rho_{1} = \rho_{2} = \rho_{\mathrm{e}}$), while in the centre, we only assumed that $\rho_{2}/\rho _{0} = 2$. The effect of the asymmetry is most intense for small $k x_{0}$, when there is most interaction between the interfaces. The panel on the right illustrates how the wide asymmetric slab becomes unstable when the interface corresponding to the highest density ratio becomes unstable. For $\rho_{1} < \rho_{2}$, the threshold corresponds to the interface with the constant density ratio (represented by the horizontal dotted line), while for $\rho_{1} > \rho_{2}$, the threshold values tend to that of the interface with variable density ratio (represented by the dot-dashed curve).

## Applications

In the previous sections, we have derived the dispersion relation for a steady slab embedded in an asymmetric environment and obtained approximate and general solutions. We now wish to discuss possible applications of this model and how it compares to previous formulations.

**Figure 9:**
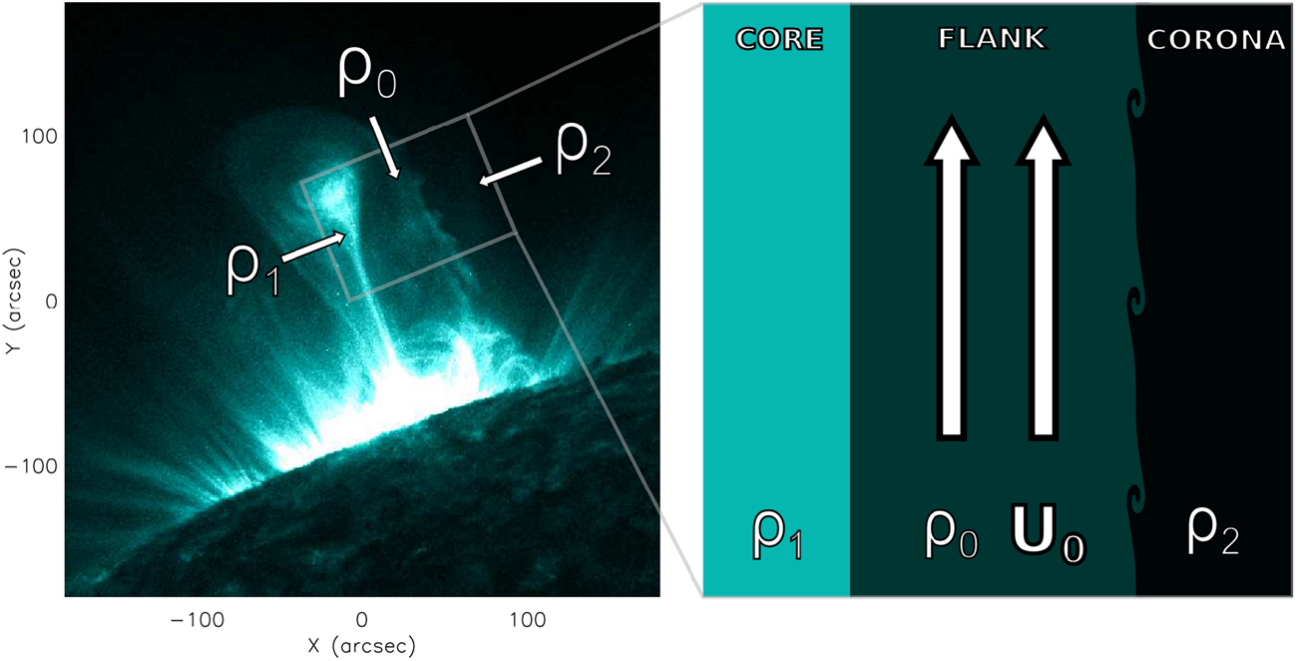
KHI detected on the flank of the CME is displayed on the *left*. The *box on the right* is a schematic representation of the unstable region. For more details about the spatial and temporal evolution of this event, see Foullon *et al.* (2011).

We primarily focus on the observations described in Foullon *et al.* (2011) of a KHI at a CME flank. The event observed by the *Atmospheric Imaging Assembly* on board the *Solar Dynamics Observatory* on November 3, 2010, was described as a series of Kelvin–Helmholtz vortices propagating on the flank of a CME. The region including the flank may be interpreted as a three-layer waveguide, with the dense CME core on one side, the CME flank in the middle, and the low-density solar corona on the other side, as in Figure 9. Since the core ejecta is much slower than the flank on the timescale of the instability, it is reasonable to approximate it as being static.

Using the parameters measured by Foullon *et al.* (2011) and Equation 11, we wish to estimate the densities of the CME core and flank in relation to the coronal background density. We begin by assuming a background Alfvén speed $v_{\mathrm{A}} = 800~\mbox{km}\,\mbox{s}^{-1}$, and sound speed $c_{0} = 0.6 v_{\mathrm{A}}$. The speeds of the ejecta flow and of the perturbations at the interface were measured to be $U_{0} = 833 \pm5~\mbox{km}\,\mbox{s}^{-1}$ and $c_{\mathrm{ph}} = 417 \pm7~\mbox{km}\,\mbox{s}^{-1}$, respectively. Using these values, we calculate the Alfvén Mach number of the flow, $M_{\mathrm{A}} \approx1.05$, and the non-dimensionalised phase speed, $\bar{c}_{\mathrm{ph}} = 0.521 \pm 0.009$. The wavenumber is measured as $k \approx0.35~\mbox{Mm}$, and the width of the shear layer is estimated to be $2 x_{0} \approx2.25 \pm1.5~\mbox{Mm}$, making $k x_{0} \approx0.394 \pm0.263$. Since $k x_{0} < 1$, there will be interactions between the boundaries of the shear layer, meaning that the density asymmetry will play an important role in the formation of the KHI.

Before we start our analysis, we must first note that when the density contrast between the three regions is such that $\min\{\bar{c}_{1}, \bar{c}_{2}\} < \min\{\bar{c}_{0} + M_{\mathrm{A}}, 1 + M_{\mathrm{A}}\}$, there exist no trapped fast modes (as is the case in Figure 6). Since we expect this to be the case, we immediately discount the fast modes. We interpret the observation as that of a slow kink mode propagating along a highly asymmetric steady slab. It has been shown by Allcock and Erdélyi (2017) that for both slow and fast modes, the transverse component of the displacement is highly sensitive to the density asymmetry. The slow mode interpretation is therefore reasonable, even though one would expect little transverse displacement in the low-beta coronal plasma.

**Figure 10:**
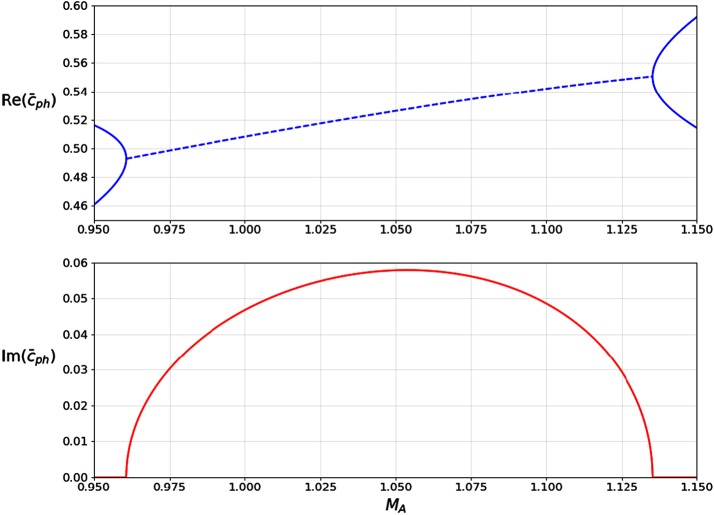
Slow kink mode plotted for $c_{0} = 0.6 v_{\mathrm{A}}$, $\rho _{1}/\rho_{0} = 1.7$, $\rho_{2}/\rho_{0} = 10^{-6}$, and $k x_{0} = 0.5$. The *upper* and *lower panels* contain the real and imaginary parts of the non-dimensionalised phase speed, respectively.

The results of the numerical analysis are presented in Figure 10, where we assumed density ratios of $\rho_{1}/\rho_{0} = 1.7$, and $\rho_{2}/\rho_{0} = 10^{-6}$. For $M_{\mathrm{A}} \approx1.05$, we obtain $\bar{c}_{\mathrm{ph}} = 0.526$, which matches the observed phase speed estimate of $\bar{c}_{\mathrm{ph}} = 0.521 + \pm0.009$. The growth rate of the instability, *i.e.* the imaginary part of $\omega$, is calculated to be $\gamma\approx0.023~\mbox{s}^{-1}$, which compares reasonably well with the observed growth rate of $\gamma= 0.05 \pm0.03~\mbox{s}^{-1}$.

We note that the choice of density ratios is significantly more sensitive on the interface separating the core from the flank. We were able to obtain values of $\bar{c}_{\mathrm{ph}}$ and $\gamma$ in close agreement with the observations for values of $\rho_{1}/\rho_{0}$ in the range ($1.6, 1.8$). On the other hand, $\rho_{2}/\rho_{0}$ may be as high as $10^{-3}$, with values lower than $10^{-6}$ having very little further effect. Our model is therefore in good agreement with the observations and estimates the density of the CME ejecta to be at least six orders of magnitude higher than the background coronal density of ${\approx}\,10^{-12}~\mbox{kg}\,\mbox{m}^{-3}$.

Our interpretation is significantly more accurate than one obtained by means of a single interface model. In such a model, one would have to assume an unrealistically low Alfvén speed in order to match the observed phase speed with a high-density contrast. Otherwise, assuming a realistic Alfvén speed $v_{\mathrm{A}} = 800~\mbox{km}\,\mbox{s}^{-1}$ would yield a density ratio of $\rho_{2} / \rho_{0} = 1/3$ between the flank and the corona, which would significantly underestimate the density of the CME. Similarly, the high-density contrast could also not be obtained from a model of a slab in a symmetric environment.

One limitation of our model is that it does not adequately explain the absence of the KHI on the inner interface, between the core and the flank. It is likely that the core is permeated by a strong magnetic field that inhibits the formation of the instability. This effect would have to be included in a more realistic interpretation.

## Conclusion

The goals of the present work are twofold: to study the effects of a steady flow on the propagation of magnetoacoustic waves in a magnetic slab in an asymmetric environment, and to examine the effects of the asymmetry on the condition for occurrence of the KHI. In order to accomplish this, we solved the dispersion relation, Equation 11, using analytical approximations and numerical schemes.

Since our analysis is only concerned with trapped mode solutions, we first obtained necessary and sufficient conditions for their existence. We then classified them as surface or body, quasi-sausage or quasi-kink modes, and obtained analytical solutions using the thin slab (Equations 13 – 16), and wide slab approximations (Equation 17).

Numerical solutions of the dispersion relation Equation 11, plotted in terms of the nondimensionalised wavenumber $k x_{0}$ for specific values of the Alfvén Mach number, $M_{\mathrm{A}}$, are presented in Figures 3 to 5. The flow causes the symmetry between forward-propagating ($\omega/ k > 0$) and backward-propagating ($\omega/ k <0$) modes to break, causing various modes to no longer be trapped. Furthermore, it causes backward-propagating modes to become forward propagating after some threshold value particular to the mode. Finally, flow speeds past a critical value will cause the KHI to occur. In terms of the solutions to Equation 11, this occurs when $\omega^{2} < 0$. The imaginary part of the solution acts as the growth rate in the time evolution of the wave, causing it to steepen (see panels (b) and (c) in Figure 1).

We wish to establish the qualitative effects of the asymmetry on the KHI in order to generalise the results of Allcock and Erdélyi (2017) on wave propagation. The authors found that asymmetry in the density profile asymmetrically modifies the amplitudes of the quasi-sausage and quasi-kink modes differently. In a symmetric slab, these modes would have anti-symmetric and symmetric amplitudes about the $z$-axis, respectively. However, the asymmetric density profile causes the quasi-sausage mode to increase in amplitude about the interface separating the interior from the lower density region, and decrease in amplitude about the other. The converse is true for the quasi-kink mode.

Considering the above, we hypothesise that for highly asymmetric density profiles and for intermediate or high values of $k x_{0}$, the slab may become asymmetrically unstable. If this is true, a quasi-sausage wave should trigger the KHI at the boundary separating the sparser region from the interior, while the converse should be true for the quasi-kink. However, a more detailed analysis of the eigenfunctions is required in order to confirm this.

Highly asymmetric systems, such as the CME flank in Foullon *et al.* (2011), are likely prone to KHIs as long as the boundaries of the slab interact. In that example, the low density of the corona stabilises the CME flank, while the high-density core destabilises it, and we observe the KHI. Because this configuration of CMEs is not uncommon, we suggest that the limited number of observations are not indicative of the number of instances of the KHI in these phenomena. Further study is needed in order to determine its prevalence. Applications of this model are in no way limited to CME flanks, even though they received much attention in this study. Any analysis of a steady configuration, whether solar or magnetospheric, that may be approximated by a slab geometry, would likely benefit from the inclusion of asymmetry.
